# Mandibular Plasmacytoma as the Initial Manifestation of Non-secretory Multiple Myeloma: A Rare Diagnostic Challenge

**DOI:** 10.7759/cureus.114506

**Published:** 2026-08-13

**Authors:** Ashok Kumar KR, Snehasree Das

**Affiliations:** 1 Department of Oral and Maxillofacial Surgery, Sri Siddhartha Academy of Higher Education, Tumkur, IND; 2 Department of Oral and Maxillofacial Surgery, Sri Siddhartha Dental College and Hospital, Tumkur, IND

**Keywords:** immunohistochemistry, mandible, multiple myeloma, osteolytic lesion, pet scan, plasma cell neoplasm, plasmacytoma

## Abstract

Plasma cell neoplasms involving the jaws are rare, with mandibular involvement representing an uncommon initial manifestation of systemic multiple myeloma (MM). Their nonspecific clinical and radiographic presentation often mimics more common odontogenic and inflammatory conditions, making early diagnosis challenging.

We report the case of an 81-year-old man who presented with pain and swelling over the left posterior mandible of two months' duration. Clinical examination revealed diffuse swelling involving the left mandibular body and angle. Orthopantomography (OPG) demonstrated a well-defined unilocular radiolucent lesion extending from the left mandibular second premolar to the retromolar region with associated cortical expansion. The histopathological examination of an incisional biopsy revealed diffuse sheets of atypical plasma cells. Initial immunohistochemistry (IHC) demonstrated CD56 positivity, and subsequent evaluation showed diffuse membranous CD138 positivity with kappa light-chain restriction and lambda negativity, confirming a monoclonal (M) plasma cell neoplasm. To differentiate a solitary bone plasmacytoma (SBP) from systemic disease, whole-body 18F-fluorodeoxyglucose positron emission tomography-computed tomography (18F-FDG PET-CT) was performed. Imaging demonstrated a hypermetabolic destructive soft tissue mass involving the left hemimandible measuring 4.6 × 3.9 cm (maximum standardized uptake value {SUVmax}: 16.5), along with multiple FDG-avid osteolytic lesions involving the frontal calvarium, left fourth rib, left iliac bone, and cervical, thoracic, and lumbar vertebrae. Bone marrow biopsy revealed marked plasma cell infiltration, and the correlation of the clinical, radiological, histopathological, immunophenotypic, and marrow findings established the diagnosis of multiple myeloma presenting as a mandibular plasmacytoma. The patient subsequently received palliative external beam radiotherapy (20 Gy in five fractions) to the mandibular lesion and was referred for systemic hematologic management.

This case underscores the importance of considering plasma cell neoplasms in the differential diagnosis of destructive mandibular lesions and highlights the indispensable role of comprehensive diagnostic evaluation, including immunohistochemistry, whole-body PET-CT, and bone marrow examination, in distinguishing localized plasmacytoma from multiple myeloma. Early recognition and multidisciplinary management are essential to ensure timely diagnosis, accurate staging, and appropriate treatment, ultimately improving patient outcomes.

## Introduction

Plasma cell neoplasms are a heterogeneous group of lymphoproliferative disorders characterized by the clonal proliferation of terminally differentiated B lymphocytes (plasma cells). They encompass a spectrum of diseases ranging from localized lesions, including solitary bone plasmacytoma (SBP) and extramedullary plasmacytoma (EMP), to disseminated systemic disease in the form of multiple myeloma (MM). Solitary plasmacytoma accounts for less than 5% of all plasma cell neoplasms and is classified as SBP when confined to the bone and EMP when arising in soft tissues [1-3]. Approximately 80%-90% of solitary plasmacytomas occur within the axial skeleton, particularly the vertebrae, ribs, pelvis, and skull, while the involvement of the jaws is distinctly uncommon [1].

Multiple myeloma is the most common plasma cell malignancy and is characterized by multifocal bone marrow infiltration by clonal plasma cells, osteolytic skeletal lesions, monoclonal (M) protein production, and end-organ damage. Although oral manifestations are reported in 8%-15% of patients with multiple myeloma, they are rarely the initial presenting feature [1,4-6]. When present, mandibular involvement may manifest as pain, swelling, tooth mobility, paresthesia, pathological fractures, or radiolucent osteolytic lesions [4,5,7]. These clinical and radiographic features are nonspecific and frequently mimic odontogenic cysts, benign and malignant odontogenic tumors, chronic osteomyelitis, metastatic tumors, lymphoma, or other primary bone malignancies, making early diagnosis particularly challenging for oral and maxillofacial surgeons, otolaryngologists, and oral pathologists.

The diagnosis of plasma cell neoplasms requires a multidisciplinary approach integrating clinical examination, radiographic findings, histopathological evaluation, immunohistochemistry (IHC), laboratory investigations, and systemic imaging. Immunohistochemical markers such as CD138 and CD56, along with the demonstration of monoclonal kappa or lambda light-chain restriction, are essential for confirming the plasma cell origin and monoclonality of the lesion. Furthermore, distinguishing solitary plasmacytoma from multiple myeloma is of paramount importance because the prognosis and treatment strategies differ significantly. Whole-body 18F-fluorodeoxyglucose positron emission tomography-computed tomography (18F-FDG PET-CT) has emerged as a valuable modality for detecting occult skeletal lesions, staging disease, and differentiating localized from disseminated plasma cell neoplasms [3,6]. Bone marrow examination remains the gold standard for confirming systemic marrow involvement and establishing the diagnosis of multiple myeloma.

The present report describes a rare case of multiple myeloma presenting initially as a mandibular plasmacytoma in an elderly male patient. The diagnosis was established through comprehensive clinicopathological correlation, sequential immunohistochemical analysis, whole-body PET-CT imaging, and bone marrow biopsy. This case highlights the diagnostic challenges associated with plasma cell neoplasms of the jaws and underscores the importance of early systemic evaluation in patients presenting with destructive mandibular lesions, emphasizing the importance of timely diagnosis that can improve patient outcomes.

## Case presentation

An 81-year-old man presented to the Department of Oral and Maxillofacial Surgery with a chief complaint of pain and swelling over the left lower back tooth region for two months. The pain was dull and continuous and had progressively worsened. The swelling had gradually increased in size over the same duration and was associated with difficulty in mastication. The patient's medical history was significant for type II diabetes mellitus and hypertension, both of which were under medical management. There was no history of trauma, fever, weight loss, or previous malignancy.

Extraoral examination revealed a diffuse swelling measuring approximately 4 × 3 cm over the left angle and body of the mandible, producing mild facial asymmetry. The swelling was firm in consistency with mild tenderness on palpation, and the overlying skin appeared normal without erythema or sinus formation. No palpable cervical lymphadenopathy was noted. Intraoral examination revealed a diffuse buccal cortical expansion extending from the mandibular left second premolar to the retromolar region. The overlying mucosa appeared intact with no evidence of ulceration. The involved teeth demonstrated tenderness on percussion, while no significant mobility was observed. Lingual cortical expansion was also appreciable on palpation.

**Figure 1:**
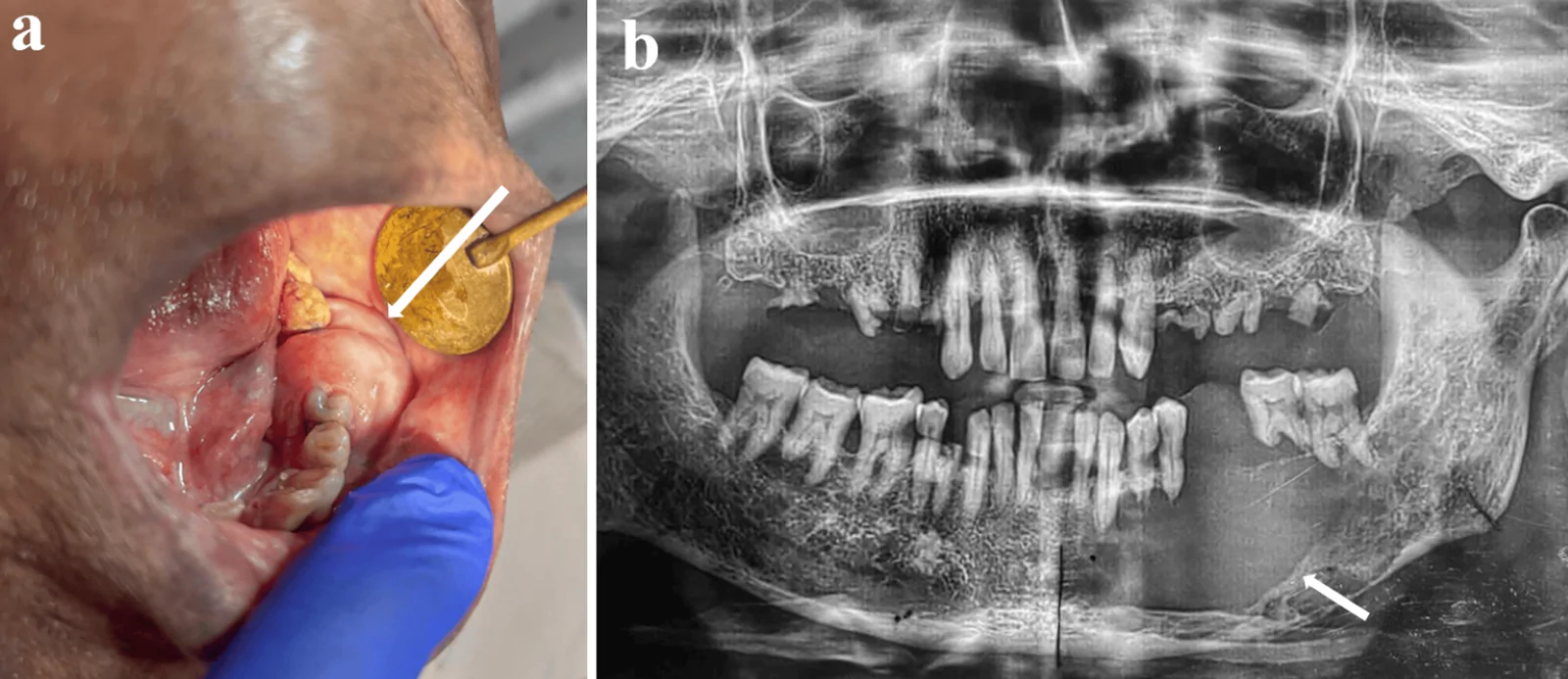
Initial clinical and radiographic presentation of mandibular plasmacytoma. (a) Intraoral photograph demonstrating a smooth, sessile swelling involving the left posterior mandibular alveolar ridge (arrow). (b) Orthopantomogram showing a well-defined unilocular osteolytic lesion extending from the left mandibular body to the angle with cortical destruction and expansion (arrow), corresponding to the site of the plasma cell neoplasm.

Orthopantomography (OPG) demonstrated a well-defined unilocular radiolucent lesion without a sclerotic margin, extending from the region of teeth 35 to 38, with the thinning and expansion of both the buccal and lingual cortical plates (Figure 1). Based on the clinical and radiographic findings, a provisional diagnosis of an odontogenic cyst or tumor was considered, with plasma cell neoplasm included in the differential diagnosis.

**Figure 2:**
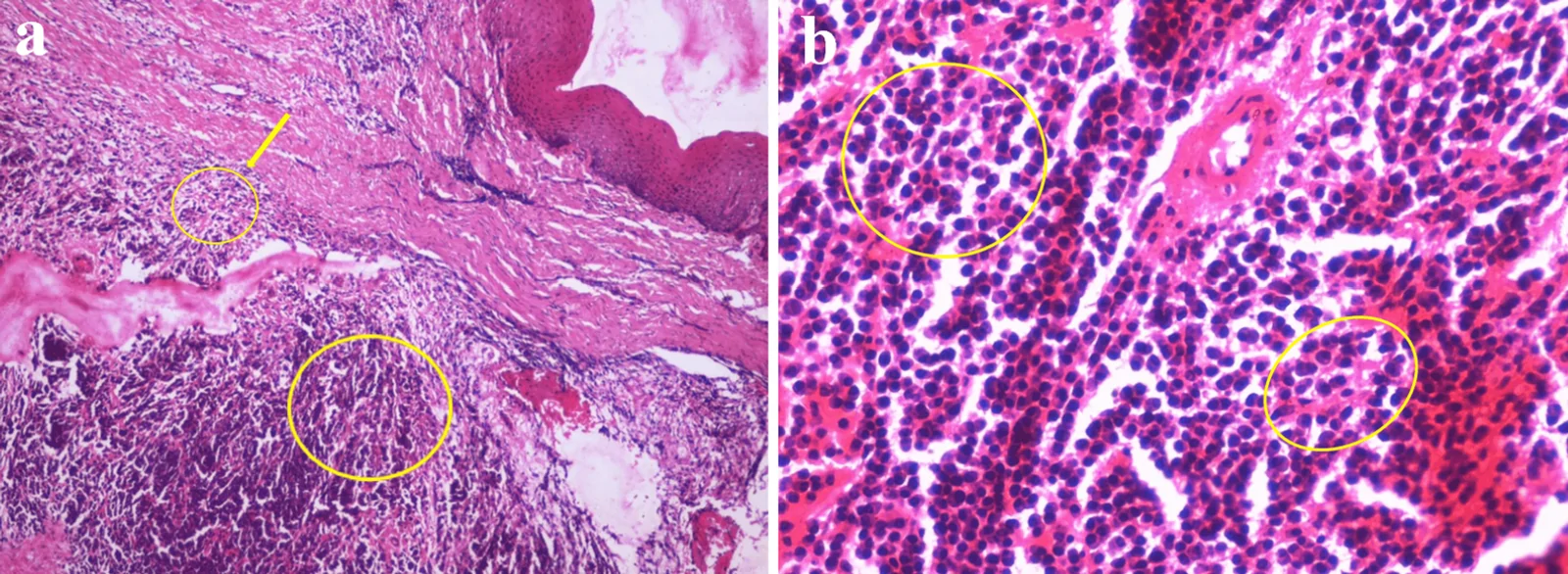
Histopathological features of plasmacytoma (hematoxylin and eosin stain). (a) Low-power view (10×) demonstrating the diffuse infiltration of the connective tissue by sheets of plasma cells beneath the overlying stratified squamous epithelium. (b) High-power view (40×) showing atypical plasma cells with eccentric nuclei, coarse "clock-face" chromatin, abundant eosinophilic cytoplasm, and occasional binucleated cells, confirming the characteristic morphology of a plasma cell neoplasm.

An incisional biopsy was performed under local anesthesia. The histopathological examination of hematoxylin and eosin-stained sections revealed stratified squamous parakeratinized epithelium overlying a highly cellular connective tissue stroma composed of diffuse sheets of plasma cells exhibiting eccentric nuclei, coarse "clock-face" chromatin, and abundant eosinophilic cytoplasm. Occasional binucleated forms and scattered lymphocytes were also identified, raising the suspicion of a plasma cell neoplasm (Figure 2).

**Figure 3:**
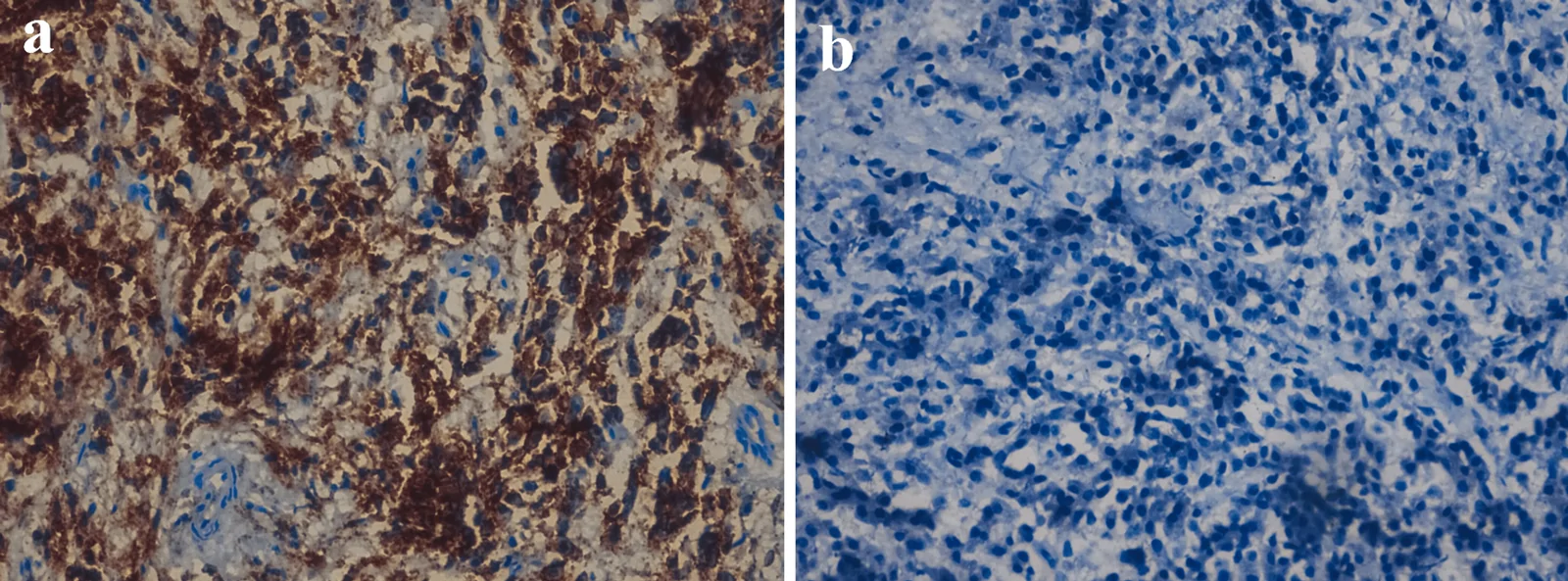
Immunohistochemical evaluation of the mandibular lesion. (a) CD56 immunostaining demonstrating strong diffuse membranous positivity in the neoplastic plasma cells, supporting the diagnosis of a plasma cell neoplasm (IHC: 400×). (b) Lambda in situ hybridization (Lambda-ISH) showing the absence of lambda light-chain expression in the neoplastic cells, indicating light-chain restriction (IHC/ISH: 400×). IHC: immunohistochemistry

Immunohistochemical analysis was subsequently undertaken to characterize the lesion. The neoplastic cells demonstrated diffuse strong membranous positivity for CD138 and positivity for CD56, confirming plasma cell differentiation (Figure 3a), and lambda in situ hybridization (Lambda-ISH) showed the absence of lambda light-chain expression in the neoplastic cells, indicating light-chain restriction (IHC/ISH: 400×) (Figure 3b).

Initial light-chain studies were inconclusive; however, repeat in situ hybridization (ISH) demonstrated kappa light-chain restriction with the absence of lambda expression, confirming a monoclonal plasma cell proliferation. The tumor cells were negative for CD3, CD20, and pan-cytokeratin (panCK), excluding lymphoid and epithelial neoplasms.

**Figure 4:**
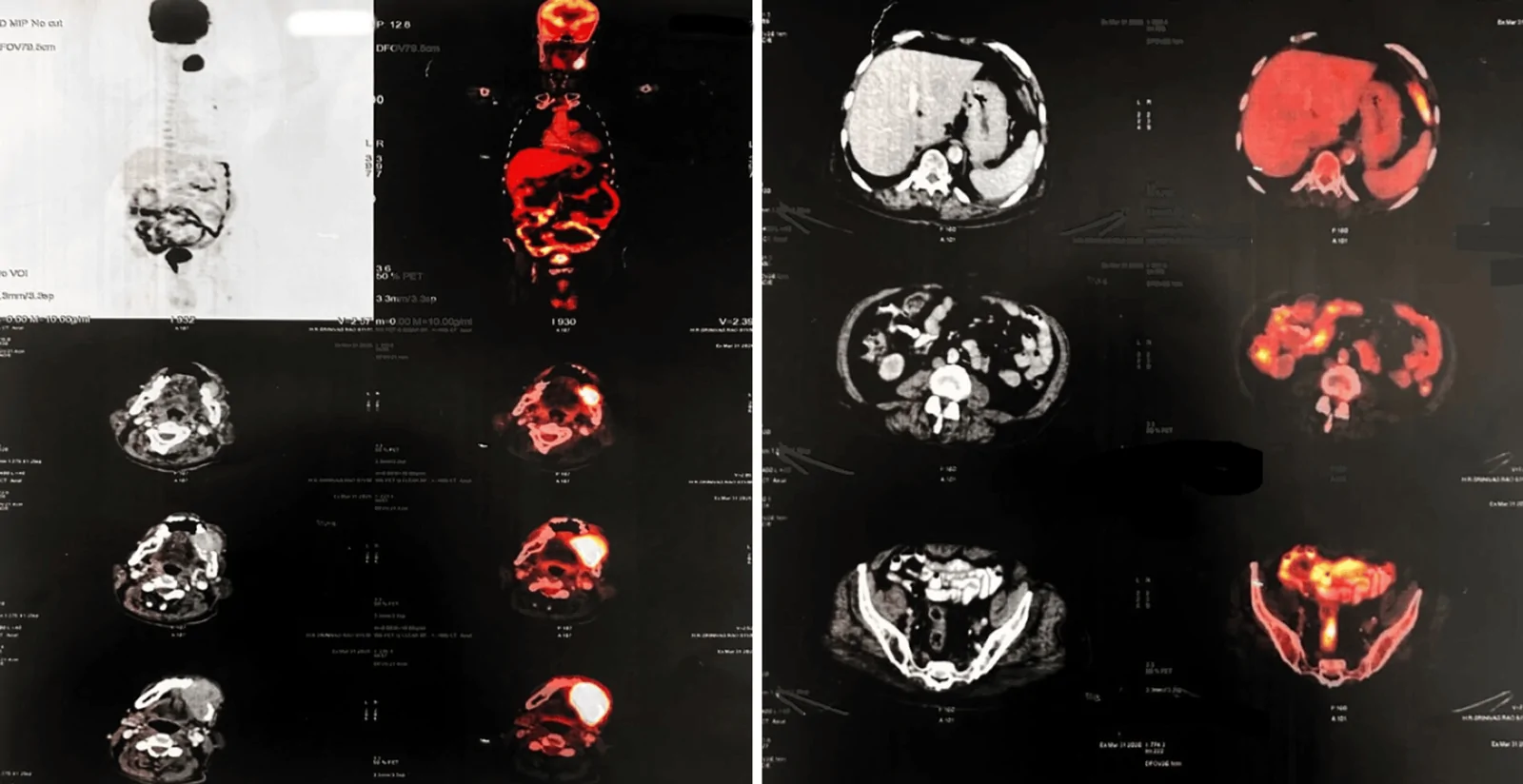
18F-FDG PET-CT findings. (a) Whole-body MIP and fused PET-CT images showing an intensely FDG-avid destructive soft tissue mass involving the left hemimandible. (b) Additional FDG-avid osteolytic lesions involving the left fourth rib, left iliac bone, and multiple vertebral bodies, confirming multifocal skeletal disease compatible with multiple myeloma. 18F-FDG PET-CT, 18F-fluorodeoxyglucose positron emission tomography-computed tomography; MIP, maximum intensity projection

To distinguish a solitary bone plasmacytoma from systemic plasma cell dyscrasia, a whole-body 18F-fluorodeoxyglucose positron emission tomography-computed tomography (18F-FDG PET-CT) was performed. PET-CT demonstrated a heterogeneously enhancing destructive soft tissue mass involving the left hemimandible, extending from the distal body to the proximal ramus and measuring 4.6 × 3.9 cm, with intense FDG uptake (maximum standardized uptake value {SUVmax}: 16.5). In addition, multiple FDG-avid osteolytic lesions were identified involving the left frontal calvarium, the shaft of the left fourth rib (SUVmax: 6.7), left iliac bone (SUVmax: 4.0), C3 vertebra (SUVmax: 3.8), thoracic vertebrae (D7-D12), and L2 vertebra (SUVmax: 3.8), suggestive of disseminated skeletal involvement (Figure 4).

Laboratory investigations revealed normocytic normochromic anemia with a hemoglobin level of 8.1 g/dL. Serum protein electrophoresis (SPEP) demonstrated hypoalbuminemia without a detectable monoclonal (M) spike. Bone marrow aspiration and trephine biopsy revealed a mildly hypercellular marrow with a marked increase in atypical plasma cells characterized by eccentric nuclei and abundant cytoplasm. The correlation of the bone marrow morphology with the histopathological, immunophenotypic, laboratory, and radiological findings established the diagnosis of multiple myeloma presenting as a mandibular plasmacytoma.

Following multidisciplinary discussion involving oral and maxillofacial surgery, hematology, radiation oncology, and pathology teams, the patient received palliative external beam radiotherapy to the mandibular lesion (20 Gy in five fractions using three-dimensional conformal radiotherapy {3D-CRT} with 6 MV photons) for local disease control and pain relief. He was subsequently referred to the hematology service for systemic chemotherapy and long-term oncologic management.

A brief about the timeline of clinical events from presentation to final diagnosis and treatment is highlighted in Table 1.

**Table 1:** Timeline of clinical events from diagnosis to management. 18F-FDG, 18F-fluorodeoxyglucose; PET-CT, positron emission tomography-computed tomography; SUVmax, maximum standardized uptake value; 3D-CRT, three-dimensional conformal radiation therapy

Date/Period	Clinical Event	Key Findings/Outcome
February 2026	Initial presentation	81-year-old man presented with pain and progressive swelling over the left posterior mandible of approximately 1-2 months' duration
March 2026	Clinical examination	Intraoral examination revealed a smooth, sessile swelling involving the left posterior mandibular alveolus with buccolingual cortical expansion
31 March 2026	Orthopantomogram (OPG)	Well-defined unilocular osteolytic radiolucency involving the left mandibular body and angle (35-38 region) with cortical expansion
Late March 2026	Incisional biopsy	Histopathology demonstrated diffuse sheets of atypical plasma cells, suggestive of a plasma cell neoplasm
March 2026	Initial Immunohistochemistry	CD56 showed diffuse positivity, confirming plasma cell lineage. Serum protein electrophoresis showed no detectable M-protein (non-secretory pattern)
31 March 2026	Whole-body 18F-FDG PET-CT	Hypermetabolic soft tissue mass involving the left hemimandible (SUVmax: 16.5) with additional FDG-avid osteolytic lesions involving the left fourth rib, left iliac bone, and multiple cervical, thoracic, and lumbar vertebrae, raising suspicion for disseminated multiple myeloma
04 April 2026	Histopathology review	Slide review favored plasma cell neoplasm and recommended additional immunohistochemical evaluation
07-10 April 2026	Extended Immunohistochemistry	Strong diffuse CD138 positivity confirmed plasma cell differentiation. Kappa light-chain positivity with lambda negativity demonstrated monoclonal plasma cell proliferation
08 April 2026	Bone marrow biopsy	Hypercellular marrow with diffuse plasma cell infiltration. Morphological and clinicopathological correlation confirmed multiple myeloma
13-17 April 2026	Palliative radiotherapy	External beam radiotherapy delivered to the left mandibular lesion (20 Gy in five fractions, 3D-CRT, 6 MV photons) for local disease control and symptomatic relief
After the completion of radiotherapy	Referral for systemic management	Patient referred to the hemato-oncology service for definitive systemic therapy for multiple myeloma and long-term follow-up

## Discussion

Plasma cell neoplasms comprise a spectrum of clonal B-cell malignancies characterized by the uncontrolled proliferation of terminally differentiated plasma cells [1,3]. These include monoclonal gammopathy of undetermined significance (MGUS), solitary bone plasmacytoma (SBP), extramedullary plasmacytoma (EMP), and multiple myeloma (MM). Solitary plasmacytomas account for less than 5% of plasma cell neoplasms, while multiple myeloma represents the most common and clinically aggressive form [1-3]. Distinguishing localized plasmacytoma from systemic disease is essential because prognosis and therapeutic strategies differ substantially.

The mandible is an uncommon site for plasma cell neoplasms, accounting for only a small proportion of reported cases [4-6]. When jaw involvement occurs, it predominantly affects the posterior mandible due to the persistence of hematopoietically active marrow. Oral manifestations are reported in approximately 8%-15% of patients with multiple myeloma; however, they are rarely the initial presenting feature. The present case is noteworthy because the patient initially sought treatment for mandibular pain and swelling, while the diagnosis of systemic multiple myeloma was established only after comprehensive clinicopathological and radiological evaluation.

Clinically, mandibular plasmacytomas often present with nonspecific features including pain, swelling, tooth mobility, paresthesia, gingival enlargement, cortical expansion, and occasionally pathological fractures [4,5,7]. These manifestations frequently resemble odontogenic cysts and tumors, chronic osteomyelitis, central giant cell lesions, metastatic tumors, lymphoma, or primary intraosseous malignancies [5,7].

Radiographically, plasmacytomas typically appear as solitary "punched-out" or well-defined osteolytic lesions without reactive sclerosis [5,7]. Conventional radiographs, including orthopantomography, may demonstrate the extent of local bone destruction but cannot reliably differentiate solitary plasmacytoma from multiple myeloma. Histopathological examination therefore remains indispensable. In the present case, hematoxylin and eosin-stained sections demonstrated diffuse sheets of atypical plasma cells exhibiting eccentric nuclei and abundant eosinophilic cytoplasm, findings characteristic of plasma cell neoplasms.

Immunohistochemistry plays a pivotal role in confirming plasma cell differentiation and establishing clonality. CD138 (syndecan-1) is regarded as the most reliable plasma cell marker, while CD56 expression is frequently observed in neoplastic plasma cells and supports the diagnosis of plasma cell myeloma [3,5]. The demonstration of monoclonal immunoglobulin light-chain restriction is particularly important in distinguishing neoplastic plasma cell proliferation from reactive plasmacytosis. In the present case, diffuse membranous positivity for CD138 together with CD56 positivity confirmed plasma cell differentiation. Initial light-chain studies were inconclusive; however, repeat in situ hybridization demonstrated kappa light-chain restriction with the absence of lambda expression, confirming monoclonality and reinforcing the diagnosis of a plasma cell neoplasm. This sequential immunophenotypic evaluation highlights the importance of additional ancillary studies when initial immunohistochemical findings are equivocal.

Once a plasma cell neoplasm is identified within the jaw, the exclusion of systemic disease becomes mandatory. The International Myeloma Working Group recommends comprehensive staging using advanced imaging modalities together with bone marrow examination [3,6]. Whole-body 18F-FDG PET-CT has become an integral component of this evaluation because of its superior sensitivity for detecting metabolically active osteolytic lesions compared with conventional skeletal surveys [3]. In the present case, PET-CT demonstrated an intensely FDG-avid destructive lesion involving the left hemimandible (SUVmax: 16.5) together with multiple FDG-avid osteolytic lesions involving the frontal calvarium, left fourth rib, left iliac bone, and cervical, thoracic, and lumbar vertebrae. These findings excluded solitary bone plasmacytoma and strongly suggested disseminated skeletal involvement.

Bone marrow biopsy remains the gold standard for confirming multiple myeloma [3,6]. The biopsy in our patient demonstrated a markedly increased population of atypical plasma cells within a mildly hypercellular marrow. The correlation of the bone marrow morphology with the histopathological findings, immunophenotypic profile, and PET-CT findings established the definitive diagnosis of multiple myeloma presenting with mandibular involvement.

A notable feature of the present case was the absence of a detectable monoclonal (M) protein on serum protein electrophoresis (SPEP) despite definitive evidence of plasma cell neoplasia. Although most patients with multiple myeloma demonstrate a measurable monoclonal immunoglobulin, approximately 1%-3% have non-secretory multiple myeloma, while some others have oligosecretory disease detectable only by sensitive assays such as serum free light-chain testing [6]. In our patient, the lack of an M-spike could have delayed the diagnosis if interpreted in isolation. However, immunohistochemistry demonstrated diffuse CD138 and CD56 positivity, with kappa light-chain restriction and absent lambda expression, confirming a monoclonal plasma cell proliferation. Furthermore, PET-CT revealed multifocal FDG-avid osteolytic lesions, and bone marrow biopsy confirmed diffuse plasma cell infiltration, establishing the diagnosis of multiple myeloma. This case emphasizes that a negative SPEP does not exclude multiple myeloma, and comprehensive clinicopathological correlation remains essential for timely diagnosis [6].

The diagnosis of multiple myeloma significantly altered the therapeutic approach in the present case. Solitary bone plasmacytoma is generally managed with definitive local radiotherapy, with or without surgical intervention, whereas multiple myeloma requires systemic therapy in addition to local treatment for symptomatic lesions [3]. Our patient received palliative external beam radiotherapy (20 Gy in five fractions) to the mandibular lesion for pain relief and local disease control, followed by referral to the hematology service for systemic chemotherapy. This multidisciplinary approach is consistent with current recommendations for the management of symptomatic multiple myeloma.

A review of the published literature indicates that mandibular involvement as the initial manifestation of multiple myeloma remains uncommon [4,5,7,8]. Most reported cases involve elderly individuals presenting with pain, swelling, tooth mobility, or paresthesia, with diagnosis established only after biopsy and systemic evaluation [4,5,7,9]. The present case differs from previously published reports because the mandibular lesion represented the first manifestation of disseminated multiple myeloma despite the absence of a detectable M-protein on serum electrophoresis. Sequential immunophenotypic evaluation with CD56, CD138, and repeat kappa/lambda ISH, combined with whole-body PET-CT and bone marrow biopsy, was critical in establishing the diagnosis.

A brief on previously reported cases of mandibular plasmacytoma and their association with multiple myeloma is provided in Table 2, highlighting the mandibular site involvement, initial clinical presentation, radiological findings, IHC findings, treatment, and outcomes.

**Table 2:** Comparison of previously reported mandibular plasmacytoma cases and their association with multiple myeloma (MM). SBP, solitary bone plasmacytoma; RT, radiotherapy; FDG, fluorodeoxyglucose; PET-CT, positron emission tomography-computed tomography; SUVmax, maximum standardized uptake value; F, female; M, male; IHC, immunohistochemistry

Author (Year)	Age/Sex	Mandibular Site	Clinical Presentation	Radiographic Findings	IHC Findings	MM at Presentation	Treatment	Outcome
Kanazawa et al. (1993) [7]	57/M	Posterior mandible	Pain and swelling	Osteolytic lesion	Plasma cell markers positive	No (SBP)	Radiotherapy	Local disease control
Pisano et al. (1997) (case series) [5]	39-79	Mandible/maxilla	Swelling and tooth mobility	Well-defined osteolytic lesions	Monoclonal plasma cells	Some patients progressed during follow-up	RT ± surgery	Variable
Bachar et al. (2008) [2]	61/M	Mandible (rare site within series)	Swelling	Osteolytic lesion	Plasma cell markers positive	No	Radiotherapy	Good local control
Goetze et al. (2015) [9]	76/F	Mandibular condyle	Pain and swelling	Osteolytic condylar lesion	Plasma cell neoplasm	Yes	Chemotherapy + bisphosphonates	Symptomatic improvement
Suryavanshi et al. (2021) [4]	65/M	Posterior mandible	Swelling and paresthesia	Unilocular osteolytic lesion	CD138+ and lambda restriction	No	Radiotherapy	Good local control
Kulkarni et al. (2021) [8]	65/F	Bilateral posterior mandible	Bilateral swelling	Expansile osteolytic lesions	Plasma cell markers positive	No	Surgery ± radiotherapy	Under surveillance
Present case	81/M	Left posterior mandible (body and ramus)	Pain, swelling, and buccolingual cortical expansion	Well-defined osteolytic lesion; FDG-avid mandibular mass (SUVmax: 16.5) with multiple skeletal lesions on PET-CT	CD56+, CD138+, kappa+, and lambda-	Yes	Palliative radiotherapy (20 Gy/five fractions), followed by systemic hematologic management	Under oncologic follow-up

Early biopsy, comprehensive immunophenotypic characterization, advanced whole-body imaging, and bone marrow examination are essential for establishing the correct diagnosis and initiating timely multidisciplinary management.

## Conclusions

Mandibular plasma cell neoplasms should not be considered isolated lesions until systemic disease has been thoroughly excluded. This case demonstrates that an apparently localized mandibular plasmacytoma may represent the first clinical manifestation of multiple myeloma, underscoring the indispensable role of comprehensive staging with immunohistochemistry, whole-body PET-CT, and bone marrow biopsy. Early diagnosis through a multidisciplinary approach enables the timely institution of systemic therapy and local disease control, thereby improving clinical outcomes. This case reinforces the need for oral and maxillofacial surgeons, otolaryngologists, oral pathologists, and hematologists to maintain a high index of suspicion when evaluating destructive mandibular lesions in elderly patients, recognize these lesions as potential indicators of underlying plasma cell dyscrasia, and ensure appropriate oncologic evaluation.
